# Comparisons of plasma and fecal pharmacokinetics of danofloxacin and enrofloxacin in healthy and *Mannheimia haemolytica* infected calves

**DOI:** 10.1038/s41598-022-08945-z

**Published:** 2022-03-24

**Authors:** Ashenafi Feyisa Beyi, Jonathan P. Mochel, Géraldine Magnin, Tyler Hawbecker, Clare Slagel, Grant Dewell, Renee Dewell, Orhan Sahin, Johann F. Coetzee, Qijing Zhang, Paul J. Plummer

**Affiliations:** 1 Department of Veterinary Microbiology and Preventative Medicine, College of Veterinary Medicine, Iowa State University, Ames, IA 50011 USA; 2 Department of Veterinary Diagnostic and Production Animal Medicine, College of Veterinary Medicine, Iowa State University, Ames, IA 50011 USA; 3 Department of Anatomy and Physiology, College of Veterinary Medicine, Kansas State University, Manhattan, KS 66502 USA; 4 College of Veterinary Medicine, Iowa State University, Ames, IA 50011 USA; 5 Center for Food Security/Public Health, College of Veterinary Medicine, Iowa State University, Ames, IA 50011 USA; 6 Nanotechnology Innovation Center of Kansas State (NICKS) and Institute of Computational Comparative Medicine, Kansas State University, Manhattan, KS 66502 USA; 7 National Institute of Antimicrobial Resistance Research and Education, Iowa State University, Ames, IA 50010 USA

**Keywords:** Pharmacology, Antimicrobials

## Abstract

Danofloxacin and enrofloxacin are fluoroquinolones (FQs) used to treat and control bovine respiratory disease (BRD) complex. While low toxicity, high bactericidal activity, and availability in single and multiple dosing regimens make them preferable, the increasing incidence of FQ-resistance in foodborne pathogens and effects on gut microbiota necessitate evaluating their pharmacokinetics (PKs). The objective of this study was to determine the exposure level of gut microbiota to subcutaneously administered FQs and compare their PKs between plasma and feces in healthy and *Mannheimia haemolytica* infected calves*.* A single dose of danofloxacin (8 mg/kg), low dose (7.5 mg/kg), or high dose (12.5 mg/kg) of enrofloxacin was administered to calves. Blood and feces were collected from calves under experimental conditions over 48 h, and FQ concentrations were measured using Ultra High-Pressure Liquid Chromatography. While moderate BRD signs were exhibited in most calves in the infected cohorts, the plasma PKs were similar between healthy and sick calves. However, the fecal danofloxacin concentration was lower in the BRD group (area under concentration–time curve [AUC_inf_], BRD median = 2627, healthy median = 2941 h*μg/mL, adj.*P* = 0.005). The dose normalized plasma and fecal danofloxacin concentrations were higher than those of enrofloxacin and its metabolite ciprofloxacin. Further, FQs had several fold higher overall concentrations in feces than in plasma in both groups. In conclusion, parenterally administered FQs expose gut microbiota to high concentrations of the antibiotics.

## Introduction

Fluoroquinolone (FQ) drugs are critically important synthetic antimicrobials used to treat infections caused by several Gram-negative bacteria, some Gram-positive bacteria, and *Mycoplasma* species. They block replication of DNA by binding to and stabilizing DNA cleavage complexes formed by DNA gyrase and topoisomerase^1,2^. In this antibiotic class, there are two crucial drugs, enrofloxacin and danofloxacin, that are preferred by veterinarians and producers to treat and control bovine respiratory disease (BRD) complex in beef cattle due to their effectiveness after a single dose administration, reducing the labor requirement^3–5^. However, there are growing concerns about using these medically important antibiotics in livestock, as some studies indicate increasing rates of FQ resistance in pathogens of public health importance. For instance, an epidemiological study conducted in feedlot cattle in multiple states of the US revealed that the prevalence of FQ-resistant *Campylobacter jejuni* has significantly increased compared to the prevalence reported in earlier studies^6^. The increase in the resistance prevalence appears to parallel the increased use of FQs in food-producing animals, as it can be deducted from the rise in the sale and distribution of FQs used in food animals since the US Food and Drug Administration (FDA) started reporting this drug in 2013^7^. Furthermore, the increased incidence of FQ resistant *C. jejuni* has led it to being listed as a public health threat by the US Centers for Disease Control and Prevention (CDC) on the second category as a “serious public health threat”, only preceded in the rank by five pathogens classified under “urgent public health threats”^8^. However, it should be taken cautiously that the correlation between the FQ uses in livestock and the rise of FQ resistance in *C. jejuni* does not necessarily confirm a causal relationship. For instance, despite the FQ exposure decreasing by 70% from 2010 to 2018, ciprofloxacin resistance continued to rise in *C. jejuni* and commensal *Escherichia coli* isolated from broilers and pigs in France^9^. Inability to establish a causal relationship between FQ use and resistance suggests the development and persistence of FQ resistance are complex, and multiple factors including fitness^10^ may play roles in it. Clonal expansion of the FQ resistant *C. jejuni* strains might also contribute to the continual increase in FQ resistance despite the decrease in the use of this antibiotic.

Fluoroquinolone antibiotics such as danofloxacin and enrofloxacin are approved in the US for use in cattle and swine, but their extra-label use has been prohibited by the FDA^11,12^. They are effective in clearing susceptible respiratory infections in animals, but they can pass from the general circulation and be accumulated in the intestinal lumen^12–14^. The elimination mechanisms of FQs involve renal and hepatic pathways, with the biliary excretion contributing to the accumulation in the intestine^2,15^. Furthermore, transepithelial secretion of FQ from the blood plays a major role in the secretion and deposition of FQ into the intestine^16,17^. It has recently been shown in pigs that enrofloxacin reached the concentration level that could reduce *E. coli* population in the intestinal lumen after being administered to two groups of pigs orally and parenterally alike^13^. Altogether, previous studies indicate that the FQ elimination mechanisms can result in the deposition of FQs in the intestinal content to the concentration level that can alter microbial diversity and induce selective pressure, leading to the emergence and spread of antimicrobial resistance (AMR)^12^.

The pharmacokinetics (PKs) of antimicrobials may be different between healthy and sick animals^18,19^. For instance, in a study conducted in ducks infected with *Pasteurella multocida,* the total amount of drug in the circulation increased, and clearance and elimination rates decreased relative to the healthy control ducks^18^. Disease and inflammation can alter drug distribution, metabolism, and elimination; however, various drugs are affected differently^19^. Thus, comparative studies of the FQ dispositions in healthy and diseased animals are vital to optimize treatment strategies.

Pharmacokinetic characteristics of antibiotics are critically important in selecting appropriate and effective drugs, adjusting dosing regimens, and enhancing antimicrobial stewardship. However, the availability of PK parameters for danofloxacin and enrofloxacin in beef cattle, particularly in *M. haemolytica* infected animals, is limited. Furthermore, studies that compare the kinetics in different dosing regimens and between plasma and fecal samples are rare. These pieces of information are crucial to minimizing the impacts of FQs on the development and spread of AMR and gut microbial diversity while maintaining optimal antimicrobial efficacy against respiratory infections.

In this study, we assessed the degree of exposure of gut microbiota to different FQs and compared the pharmacokinetics of danofloxacin and enrofloxacin (and its active metabolite ciprofloxacin) in healthy control and *M. haemolytica* infected calves. In addition, we evaluated the PK differences between plasma and feces as well as between low and high doses of enrofloxacin.

## Materials and methods

### Animal study 1

Twenty two-month-old calves weighing between 54 and 93 kg with no prior history of antibiotic exposure were used for this study. These Holstein genetic calves (26 males; 4 females) were sourced from a dairy farm in Iowa. They were group housed at the Livestock Infectious Disease Isolation Facility (LIDIF, Biosafety level 2) of Iowa State University for 28 days. Each room had an independent airflow system and was maintained at 20–21 °C. The logistic was arranged in the way that it prevented cross-contamination of the rooms. The calves were fed mixed grass hay and a premixed calf starter with unlimited water provision during the study. Serious health issues that required administration of antibiotics did not happen during the course of the study. Ten calves in one group (BRD group) were challenged with *M. haemolytica* suspended in PBS (10 mL per calf, ~ 3 × 10^9^ CFU/mL) to induce BRD by trans-tracheal injection using a sterile intravenous catheter according to the method explained in our previous publication^20^. The other ten calves (control/healthy group) were kept in a separate room with an independent airflow system. The *M. haemolytica* strain used in this study was originally isolated from the lung of a dead calf diagnosed with pneumonic mannheimiosis at Iowa State University’s Veterinary Diagnostic Laboratory. As described in our previous publication^20^, the isolate was recovered on MH agar from frozen glycerol stock (− 80 °C), followed by fresh culture preparation by sub-passaging on new MH agar plates (overnight incubation at 37 °C). Cells were harvested in sterile saline, centrifuged at 3000 rcf for 20 min, and the pellet was suspended in fresh saline to obtain a suspension of OD_600_ of 2.0 (~ 1.0 × 10^9^ CFU/mL), which was administered to the calves.

In the following week, the calves in the BRD group were monitored for BRD signs, including elevated body temperature, eye and nasal discharges, ear droop or head tilting, cough, and changes in breathing, eating, and ambulation. On the eighth day, both groups were administered a single dose of danofloxacin (8 mg/kg body weight, ADVOCIN™, *danofloxacin mesylate*, Pfizer Animal Health) in the neck, subcutaneously. The animal study was described in more detail in our recent publication^21^.

### Animal study 2

Twenty-eight Holstein male calves in the age of three to four months with weights ranging from 73 to 135 kg were obtained from a farm in Wisconsin. They were divided randomly into four groups and kept in four separate rooms with an independent airflow system. The logistic was arranged in the way that it prevented cross-contamination of the rooms during this study as well. The calf management was similar with study 1 once they arrived at the LIDIF. These calves were not exposed to any antibiotics before being enrolled into this study. *M. haemolytica* suspended in PBS (20 mL per calf, 5 × 10^8^ CFU/mL) was inoculated via trans-tracheal injection using a catheter to induce BRD in calves in two of the four groups. The *M. haemolytica* inoculum was prepared in the same way as study 1. They were followed in the subsequent week for the exhibition of BRD signs. Two dosage levels of enrofloxacin (low dose = 7.5 mg/kg, high dose = 12.5 mg/kg, BAYTRIL™ 100, Bayer Animal Health, Shawnee Mission, KS) were administered in a single-dose regimen to all calves in the neck subcutaneously after eight days of *M. haemolytica* challenge. In this study, there were four groups of calves: low-dose control, low-dose BRD, high-dose control, and high-dose BRD. The animal study was detailed in our recent publication^22^.

The ARRIVE guidelines were followed in conducting these experiments. Both animal studies were approved by Iowa State University Institutional Animal Care and Use Committee (IACUC-18-372), and we followed those prior approved protocols during the trials. At the end of the studies, the calves were euthanized according to the American Veterinary Medical Association guidelines using a penetrating captive bolt gun^23^. All other procedures involving animals were also carried out in accordance with relevant guidelines and regulations. The study calves were monitored for the BRD development for one week after *M. haemolytica* inoculation, and the baseline parameters were taken two days before the challenge. The categorization of the calves into BRD positive or negative was conducted based on the scoring system developed at UC Davis for dairy calves^24^. In this scoring system, typical clinical signs of BRD are provided with scores; if the sum of the scores of the clinical signs demonstrated by a calf is greater than or equal to five, that calf can be considered as BRD positive.

### Blood draw and plasma harvest

Blood was collected from the jugular vein at times 0, 0.25, 0.5, 1, 2, 4, 6, 8, 12, 24, 36, and 48 h after danofloxacin and enrofloxacin administration. A catheter was placed one day before the FQ injection to draw blood. Briefly, one side of the neck was shaved and subsequently cleaned with three alternating scrubs of isopropyl alcohol and chlorhexidine, followed by injection of 3 mL of lidocaine subcutaneously at the site where the catheter was to be inserted. Then, the catheter was inserted and fixed to the skin to prevent its removal. It was flushed with heparinized saline, and an intravenous extension line and injection cap were attached. Finally, an adhesive bandage was put around the neck of the calf to protect the catheter. These catheters were dedicated to sample collection and no antibiotic was administered through them. During sample collection, the calves were restrained with head halters and the catheter was flushed with heparinized saline. First, “waste” blood was pulled to clear the catheter of any residual saline, and then sample blood was drawn with a syringe and transferred to a 10 mL green cap test tube (heparinized) followed by flushing the catheter again using the saline. The drawn blood samples were put on ice until centrifugation, which was conducted within 15 min of sampling. They were spun down (1300 rcf for 15 min), then about 2 mL plasma was transferred in duplicates to pre-labeled cryovial tubes and stored at − 20 °C and transferred to the research laboratory to be stored at − 80 °C until assayed.

Fecal samples were collected at time points 0, 1, 2, 4, 6, 8, 12, 24, 36, and 48 h following the antibiotic treatment directly from the rectum into sterile screw-capped 50 mL universal tubes. To ensure aseptic collection of the feces, gloves were changed between every animal to induce defecation and add the feces to the tubes. The fecal samples were kept on ice until transferred to the laboratory within an hour of collection, where they were stored at – 80 °C. Both the blood and fecal samples were shipped to Kansas State University on dry-ice for chemistry analyses.

### Determination of fluoroquinolones in plasma

To determine the concentrations of FQs (ciprofloxacin, danofloxacin, and enrofloxacin) in plasma, an LC–MS/MS method was developed. The plasma samples were cleaned before the analysis using solid-phase extraction. They were analyzed by Ultra High-Pressure Liquid Chromatography (UPLC) and detected using positive electrospray ionization (ESI) with multiple reaction monitoring (MRM). In this method, deuterated analogs of the fluoroquinolones were used as internal standards. Before each analysis, quality controls were prepared by spiking untreated bovine plasma with the fluoroquinolones at the following levels: 10 (QC1), 100 (QC2), 400 (QC3) and 4000 ng/mL (QC4). For calibration, plasma was spiked with increasing concentration of the FQs and were processed similarly as the samples. The response (analyte over internal standard) was plotted against the concentration (ng/mL). The best fit was selected for calibration of FQs using linear regression with a weighting factor of 1/x and the coefficient of correlation at least > 0.99. Accuracy was measured using three replications per four concentrations (10, 100, 400, and 4000 ng/mL) spiked into untreated bovine plasma. The lower limit of quantification (LLOQ) was determined to be at 0.01 μg/mL for all fluoroquinolones in the conditions used. The limit of detection (LOD) was determined to be at 5 ppb with at least a signal over noise ratio of 3:1.

### Determination of fluoroquinolones in feces

An approach was developed to measure the concentration of danofloxacin and enrofloxacin, including its active metabolite ciprofloxacin, in feces by LC–MS/MS. About one gram of feces was extracted with acetonitrile using QuECHERs salt in the presence of EDTA. UHPLC was used to analyze these antibiotics. Further, they were detected by ESI using multiple reaction monitoring (MRM). As mentioned above for plasma, deuterated analogs of the FQs were used for the internal standards. Quality controls were prepared before each analysis by spiking untreated/control bovine feces (1 g) with the FQs at QC3 (2 μg/g), QC2 (5 μg/g), and QC1 (10 μg/g) and were extracted in a similar way as the samples. Because of the high concentration of FQs in the fecal samples and the high dilutions required, external standard calibration was employed. The internal standard was used after samples extraction and dilution to account for the variation of the instrument ionization throughout the run. The calibration was conducted in the same way as that of FQs in the plasma by fitting linear regression with a weighting factor of 1/x and the coefficient of correlation at least > 0.99. Replicate analysis of known amounts of FQs spiked into untreated bovine feces was used to calculate the recovery. The LLOQ was determined to be at 0.01 μg/mL in the extract which corresponds to 0.475 μg/g in feces for all fluoroquinolones in the conditions used. The LOD was determined to be at 5 ng/mL in the extract or 0.238 μg/g in feces with at least a signal over noise ratio of 3:1.

### Determination of pharmacokinetic parameters

A commercial software, PKanalix (PKanalix, Monolix Suite 2019R1, Lixoft, France)^25^, was used to compute PK parameters using a statistical moments approach, which included area under the plasma/fecal concentration versus time curve extrapolated to infinity (AUC_inf_), area under the plasma/fecal concentration versus time curve from dosing to the last sampling time point (AUC_0~48_), area under the plasma/fecal concentration versus time curve from the time of dosing to the last measurable positive concentration (AUC_last_), area under the first moment curve extrapolated to infinity (AUMC_inf_), area under the first moment curve from dosing to the last measurable concentration (AUMC_last_), apparent clearance (CL/F), maximum observed drug concentration (C_max_), mean residence time from the time of dosing to the last measurable concentration (MRT_last_), time to the maximum concentration (T_max_), elimination/terminal half-life (T_1/2_λz), elimination rate (λz), and apparent volume of distribution associated with the terminal phase (Vz/F). Using the non-compartmental analysis (NCA), the log-linear trapezoidal method was used to calculate AUC in PKanalix. These parameters were presented as mean ± SD, and graphs were plotted using mean ± SE (standard error). These parameters were compared between different study groups using non-parametric pairwise comparison statistical tests; the Mann–Whitney test was used to compare two groups. To compare the BRD scores between BRD and healthy calves, Fisher’s exact test was used. A *P* ≤ 0.05 was considered significantly different unless stated otherwise.

## Results

### Bovine respiratory disease induction

Using the BRD Scoring information system developed by UC Davis, calves were categorized into BRD positive and negative^24^. In study 1, none of the calves showed BRD before *M. haemolytica* inoculation; however, after the challenge, eight out of the ten calves in the cohort had BRD score ≥ 5; except for one, the seven others had BRD positive score at least three times during the one week of monitoring. In the control group, only one calf had a BRD score ≥ 5, and the difference with the BRD group was significant (*P* = 0.037). In study 2, the calves challenged with *M. haemolytica* did not show BRD signs before the inoculation. In the group that received low dose enrofloxacin, five of the seven calves had the BRD score ≥ 5 at least once, and in the group administered the high dose, all seven calves had a score ≥ 5 at least once. In the control groups, three calves in the low-dose healthy and three calves in high-dose healthy had the BRD score ≥ 5. The difference between high dose BRD and healthy groups was marginally significant (*P* = 0.070), while the difference between the low dose BRD and healthy groups were insignificant (*P* = 0.592). However, the BRD signs were observed less frequently in the control groups compared to the *M. haemolytica* infected groups in both low and high dose cohorts. In general, the BRD score ≥ 5 was more frequently observed in the *M. haemolytica* challenged groups than the control calves, indicating the BRD was induced, but the severe disease did not develop in most calves. Furthermore, lung lesions were observed in six calves in the BRD group while only two calves had lesions in the control group of study 1. In study 2, three calves in each low and high dose BRD groups and one calf in the respective control groups had lung lesions. The lungs lesions were more extensive in the BRD groups than the control healthy groups.

### Pharmacokinetics of fluoroquinolones in plasma

Danofloxacin was detected in plasma between the time 0.25 and 24 h, while it was detected in the feces through the last sampling (i.e., 48 h post antibiotic injection). Similarly, enrofloxacin and ciprofloxacin were detected between the time 0.25 h and 48 h in plasma and between 1 and 48 h in the feces. The number of plasma and fecal samples positive for enrofloxacin and ciprofloxacin varied in the first few and the last sampling time points. For instance, enrofloxacin was detected in all plasma samples, while ciprofloxacin was detected in only six of the seven calves at 0.25 h.

**Figure 1:**
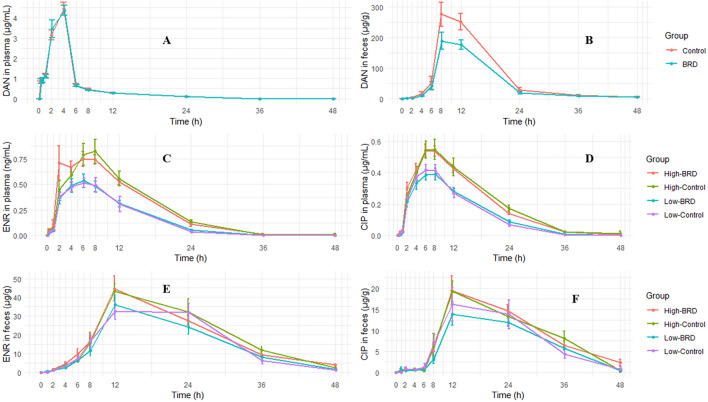
Concentration versus time plot of danofloxacin (**A** DAN in plasma, **B** DAN in feces), enrofloxacin (**C** ENR in plasma, **E** ENR in feces) and ciprofloxacin (**D** CIP in plasma, **F** CIP in feces) in plasma and feces. The concentrations depicted in the graphs were the mean of all calves in a group at a given sampling time with the standard error (mean ± SE). None of the considered PK parameters were significantly different between healthy and BRD calves in plasma data, while some of the PK parameters derived from the fecal data (i.e., **B**) were significantly different. *Groups* study 1 (Panels **A** and **B**): Control—healthy calves injected danofloxacin (DAN, 8 mg/kg, n = 10) and BRD—calves infected with *M. haemolytica* and injected danofloxacin (DAN, 8 mg/kg, n = 10); study 2 (**C**–**F**): High-BRD—calves infected with *M. haemolytica* and injected high dose enrofloxacin (ENR, 12.5 mg/kg, n = 7), High-Control—healthy calves injected high dose enrofloxacin (ENR, 12.5 mg/kg, n = 7), Low-BRD—calves infected with *M. haemolytica* and injected low dose enrofloxacin (ENR, 7.5 mg/kg, n = 7), and Low-Control—healthy calves injected low dose enrofloxacin (ENR, 7.5 mg/kg, n = 7).

The plasma and fecal concentration–time profiles of danofloxacin, enrofloxacin, and ciprofloxacin are presented in Fig. 1. This figure shows (1) absence of significant concentration differences between healthy and BRD groups in plasma at any time points; (2) presence of a significant concentration difference between healthy and BRD groups for danofloxacin in feces at sampling times 8 h and 12 h (higher in healthy group); (3) existence of a significant plasma concentration difference between low and high dose groups in the enrofloxacin treated calves.

The plasma maximum and total concentration of danofloxacin (C_max_ = 4.6 ± 1.0, AUC_last_ = 21.0 ± 4.1) were significantly higher than that of enrofloxacin (low dose: C_max_ = 0.6 ± 0.1, AUC_last_ = 6.5 ± 1.7; high dose: C_max_ = 0.8 ± 0.2, AUC_last_ = 10.3 ± 2.7) and ciprofloxacin (low dose: C_max_ = 0.39 ± 0.084, AUC_last_ = 5.2 ± 0.9; high dose: C_max_ = 0.7 ± 0.08, AUC_last_ = 10.6 ± 1.8). The dose normalized plasma concentration (C_max_/Dose and AUC_inf_/Dose) of danofloxacin was many fold higher than that of enrofloxacin and danofloxacin (Tables 1, 2, and 3). Similarly, the time spent to reach the peak concentration (T_max_) in the plasma was faster, and the terminal half-life (t_1/2_λz) was more extended in the danofloxacin group than in the other groups (Tables 1, 2, and 3). Furthermore, danofloxacin had a slower elimination rate (λz = 0.09 ± 0.01) as opposed to enrofloxacin (low-dose: λz = 0.18 ± 0.03, high-dose: λz = 0.13 ± 0.04) and ciprofloxacin (low-dose: λz = 0.12 ± 0.02, high-dose: λz = 0.15 ± 0.01). Conversely, the apparent plasma clearance (CL/F), mean residence time to the last measurable concentration (MRT_last_), and apparent volume of distribution (Vz/F) were significantly higher in the enrofloxacin administered groups than in the danofloxacin group. In addition, most of the considered PK parameters showed significant differences between low and high-dose groups for both enrofloxacin and ciprofloxacin (Tables 2 and 3). However, the AUC_inf_/Dose (high dose: 0.9 ± 0.2, low dose: 0.9 ± 0.2) and C_max_/Dose (high dose: 0.06 ± 0.02, low dose: 0.08 ± 0.02) were not significantly different between low and high dose groups for enrofloxacin. For ciprofloxacin, C_max_/Dose was significantly higher in the high dose group (0.06 ± 0.007) than in the low dose group (0.05 ± 0.008, adj. *P* = 0.027), while AUC_inf_/Dose (high dose: 0.9 ± 0.2, low dose: 0.7 ± 0.1) was not different; this difference might be attributed to the high variability of AUC compared to C_max_.

**Table 1:** The plasma pharmacokinetic parameters of danofloxacin administered to calves subcutaneously (8 mg/kg body weight, ten animals in each group) computed by a non-compartmental (i.e., statistical moments) analysis.

Parameters*	Group	Minimum	Median	Maximum	Adjusted P-value**
AUC_inf_ (h × μg/mL)	Control	13.3	23.0	29.9	0.968
	BRD	12.8	22.8	27.0	
AUC_inf_/Dose	Control	1.66	2.87	3.73	0.968
	BRD	1.60	2.85	3.37	
AUC_0~48_ (h × μg/mL)	Control	12.7	22.2	29.2	0.905
	BRD	12.6	21.5	26.2	
AUC_0~48_ /Dose	Control	1.61	2.77	3.65	0.9682
	BRD	1.57	2.69	3.28	
AUC_last_ (h × μg/mL)	Control	12.5	21.4	28.3	0.842
	BRD	12.2	20.9	25.6	
AUMC_inf_ (h^2^ × μg/mL)	Control	92.9	160.5	200.5	0.905
	BRD	86.0	141.6	196.0	
AUMC_last_ (h^2^ × μg/mL)	Control	64.3	113.3	147.7	0.842
	BRD	64.3	111.6	128.8	
Cl/F (1/h)	Control	0.27	0.35	0.60	0.935
	BRD	0.30	0.35	0.62	
C_max_ (μg/mL)	Control	2.5	4.8	5.6	0.838
	BRD	2.4	4.6	6.1	
C_max_ /Dose	Control	0.3	0.6	0.7	0.838
	BRD	0.3	0.58	0.8	
T_1/2_ λz (h)	Control	7.5	8.2	8.8	0.842
	BRD	6.5	7.7	10.6	
λz (1/h)	Control	0.08	0.08	0.09	0.838
	BRD	0.07	0.09	0.11	
MRT_inf_ (h)	Control	6.3	7.0	7.7	0.356
	BRD	5.6	6.3	8.6	
MRT_last_ (h)	Control	4.9	5.2	5.5	0.497
	BRD	4.4	5.1	5.8	
T_max_ (h)	Control	4.0	4.0	4.0	0.192
	BRD	2.0	4.0	4.0	
Vz/F (L)	Control	2.9	4.3	7.2	0.905
	BRD	3.1	4.0	6.7	

*PK-parameters area under the curve from the time of dosing extrapolated to infinity (AUC_inf_), area under the curve from the time of dosing to the last sampling time point (AUC_0~48_), area under the curve from the time of dosing to the last measurable positive concentration (AUC_last_), area under the first moment curve extrapolated to infinity (AUMC_inf_), area under the first moment curve from dosing to the last measurable concentration (AUMC_last_), apparent clearance (CL/F), maximum observed concentration (C_max_), mean residence time from the time of dosing to the last measurable concentration (MRT_last_), time to the maximum concentration (T_max_), terminal half-life (T_1/2_λz), elimination rate (λz), and apparent volume of distribution associated with the terminal phase (Vz/F). *Groups* control—healthy control calves, and BRD—*M. haemolytica* infected calves.

**Mann–Whitney test was used to compare the difference between the control and BRD groups.

**Table 2:** The plasma pharmacokinetics of enrofloxacin administered to calves subcutaneously (low dose 7.5 mg/kg, high dose 12.5 mg/kg body weight, 14 animals in each healthy and BRD combined group) computed by a non-compartmental model (i.e., statistical moments) analysis.

Parameters*	Dose	Minimum	Median	Maximum	Adjusted P-value**
AUC_inf_ (h × μg/mL)	High	5.5	11.8	12.8	0.010
	Low	4.8	6.3	10.4	
AUC_inf_/Dose	High	0.44	0.94	1.03	0.270
	Low	0.64	0.84	1.38	
AUC_0~48_ (h × μg/mL)	High	5.4	11.7	12.9	0.000
	Low	4.8	6.3	10.4	
AUC_0~48_/Dose	High	0.43	0.94	1.03	0.408
	Low	0.65	0.84	1.38	
AUC_last_ (h × μg/mL)	High	5.4	11.2	12.8	0.000
	Low	4.7	6.1	10.3	
AUMC_last_ (h^2^ × μg/mL)	High	60.7	117.2	128.2	0.000
	Low	42.1	49.8	99.6	
Cl/F (1/h)	High	0.97	1.06	2.29	NA
	Low	0.72	1.20	1.55	
C_max_ (μg/mL)	High	0.39	0.85	1.0	NA
	Low	0.41	0.6	0.84	
C_max_/Dose	High	0.03	0.07	0.08	NA
	Low	0.05	0.08	0.11	
T_1/2_ λz (h)	High	3.9	4.5	8.0	NA
	Low	3.1	3.8	4.8	
λz (1/h)	High	0.09	0.14	0.18	NA
	Low	0.14	0.18	0.22	
MRT_inf_ (h)	High	10.0	10.7	14.6	NA
	Low	8.19	9.32	10.0	
MRT_last_ (h)	High	9.8	10.3	11.3	NA
	Low	7.8	8.8	9.7	
T_max_ (h)	High	2.0	6.0	6.0	NA
	Low	4.0	6.0	6.0	
Vz/F (L)	High	5.5	9.1	17.2	NA
	Low	5.0	6.4	9.1	

*PK-parameters area under the curve from the time of dosing extrapolated to infinity (AUC_inf_), area under the curve from the time of dosing to the last sampling time point (AUC_0~48_), area under the curve from the time of dosing to the last measurable positive concentration (AUC_last_), area under the first moment curve from dosing to the last measurable concentration (AUMC_last_), apparent clearance (CL/F), maximum observed concentration (C_max_), mean residence time from the time of dosing to the last measurable concentration (MRT_last_), time to the maximum concentration (T_max_), terminal half-life (T_1/2_λz), elimination rate (λz), and apparent volume of distribution associated with the terminal phase (Vz/F). *NA* not applicable, the PK linearity does not break in the dose range of 7.5–12.5 mg/kg.

**Mann–Whitney test was used to compare the difference between the high dose and low dose groups.

**Table 3:** The plasma pharmacokinetics of ciprofloxacin in calves administered enrofloxacin subcutaneously (low dose 7.5 mg/kg, high dose 12.5 mg/kg body weight, 14 animals in each healthy and BRD combined group) computed by a non-compartmental model (i.e., statistical moments) analysis.

Parameters*	Dose	Minimum	Median	Maximum	Adjusted P-value**
AUC_inf_ (h × μg/mL)	High	9.5	9.9	12.9	0.000
	Low	4.1	5.7	6.7	
AUC_inf_/Dose	High	0.76	0.79	1.03	0.288
	Low	0.54	0.77	0.89	
AUC_0~48_ (h × μg/mL)	High	9.5	9.8	12.9	0.000
	Low	4.0	5.7	6.3	
AUC_0~48_/Dose	High	0.76	0.79	1.03	0.260
	Low	0.53	0.76	0.84	
AUC_last_ (h × μg/mL)	High	9.4	9.7	12.7	0.000
	Low	3.8	5.5	6.2	
AUMC_last_ (h^2^ × μg/mL)	High	110.4	113.8	161.5	0.000
	Low	31.8	53.7	69.7	
C_max_ (μg/mL)	High	0.68	0.69	0.83	0.001
	Low	0.31	0.40	0.49	
C_max_/Dose	High	0.05	0.06	0.07	0.027
	Low	0.04	0.05	0.07	
T_1/2_ λz (h)	High	4.3	4.9	4.9	NA
	Low	4.6	5.8	7.5	
λz (1/h)	High	0.14	0.14	0.16	NA
	Low	0.09	0.12	0.15	
MRT_inf_ (h)	High	12.2	12.3	13.1	NA
	Low	10.3	12.0	14.1	
MRT_last_ (h)	High	11.7	11.8	12.7	NA
	Low	8.5	10.2	12.0	
T_max_ (h)	High	6.0	8.0	8.0	NA
	Low	4.0	6.0	8.0	

*PK-parameters area under the curve from the time of dosing extrapolated to infinity (AUC_inf_), area under the curve from the time of dosing to the last sampling time point (AUC_0~48_), area under the curve from the time of dosing to the last measurable positive concentration (AUC_last_), area under the first moment curve from dosing to the last measurable concentration (AUMC_last_), maximum observed concentration (C_max_), mean residence time from the time of dosing to the last measurable concentration (MRT_last_), time to the maximum concentration (T_max_), and terminal half-life (T_1/2_λz), elimination rate (λz). *NA* not applicable, the PK linearity does not break in the dose range of 7.5–12.5 mg/kg.

**Mann–Whitney test was used to compare the high and low dose groups.

### Pharmacokinetics of fluoroquinolones in feces

The PK parameters of danofloxacin, enrofloxacin, and ciprofloxacin in feces are presented in Tables 4, 5 and 6. The concentration of danofloxacin (median AUC_last_: healthy 2893 h × μg/mL, sick 2548 h × μg/mL, adj. *P* = 0.005) was significantly higher in the healthy group than in the BRD group. Similarly, the peak concentration was also higher in the healthy group. However, the terminal half-life, the elimination rate, and the time to peak concentration were similar between the two groups. Compared to the plasma samples, the fecal samples had more than 100, 70, and two-fold higher in the total concentration (AUC_las_), maximum concentration (C_max_), and time to the peak concentration (T_max_), respectively.

**Table 4:** The fecal pharmacokinetic parameters of danofloxacin administered to calves subcutaneously (8 mg/kg body weight) computed by a non-compartmental model (i.e., statistical moments) analysis.

Parameters*	Group	Minimum	Median	Maximum	Adjusted P-value**
AUC_inf_ (h × μg/mL)	Control	2857	2941	4646	0.013
	BRD	1857	2627	3398	
AUC_inf_/Dose	Control	357	368	581	0.013
	BRD	232	328	425	
AUC_0~48_ (h × μg/mL)	Control	2810	2893	4402	0.005
	BRD	1772	2548	3324	
AUC_0~48_/Dose	Control	351	362	550	0.005
	BRD	222	319	416	
AUC_last_ (h × μg/mL)	Control	2810	2893	4402	0.005
	BRD	1772	2548	3324	
AUMC_last_ (h^2^ × μg/mL)	Control	35,519	36,582	86,372	0.043
	BRD	31,003	39,576	48,148	
C_max_ (μg/mL)	Control	378	382	435	0.008
	BRD	125	236	348	
C_max_/Dose	Control	47	48	54	0.008
	BRD	16	30	43	
T_1/2_ λz (h)	Control	9.7	10.8	11.2	0.315
	BRD	9.2	9.6	10.1	
λz (1/h)	Control	0.06	0.06	0.07	0.307
	BRD	0.07	0.07	0.08	
MRT_inf_ (h)	Control	13.5	13.5	21.8	0.013
	BRD	15.5	17.5	19.5	
MRT_last_ (h)	Control	12.6	12.7	19.6	0.104
	BRD	14.5	16.0	17.5	
T_max_ (h)	Control	8	8	12	0.681
	BRD	8	10	12	

*PK-parameters area under the curve from the time of dosing extrapolated to infinity (AUC_inf_), area under the curve from the time of dosing to the last sampling time point (AUC_0~48_), area under the curve from the time of dosing to the last measurable positive concentration (AUC_last_), area under the first moment curve from dosing to the last measurable concentration (AUMC_last_), maximum observed concentration (C_max_), mean residence time from the time of dosing to the last measurable concentration (MRT_last_), time to the maximum concentration (T_max_), terminal half-life (T_1/2_λz), and elimination rate (λz). *Groups* control—healthy control calves, and BRD—*M. haemolytica* infected calves.

**Mann–Whitney test was used to compare the control and BRD groups.

**Table 5:** The fecal pharmacokinetics of enrofloxacin administered to calves subcutaneously (low dose 7.5 mg/kg, high dose 12.5 mg/kg body weight, 14 animals in each healthy and BRD combined group) computed by a non-compartmental model (i.e., statistical moments) analysis.

Parameters*	Group	Minimum	Median	Maximum	Adjusted P-value**
AUC_inf_ (h × μg/mL)	High	794	1285	1354	0.008
	Low	615	761	819	
AUC_0~48_ (h × μg/mL)	High	775	1261	1330	0.085
	Low	590	754	809	
AUC_last_ (h × μg/mL)	High	775	1261	1330	0.085
	Low	590	754	809	
AUMC_last_ (h^2^ × μg/mL)	High	13,417	24,754	27,196	0.031
	Low	11,370	13,388	16,395	
C_max_ (μg/mL)	High	51.0	54.0	77.0	0.129
	Low	27.7	38.0	46.2	
T_1/2_ λz (h)	High	5.5	6.5	7.3	NA
	Low	5.0	6.8	8.4	
λz (1/h)	High	0.10	0.11	0.13	NA
	Low	0.08	0.10	0.14	
MRT_inf_ (h)	High	18.3	19.3	22.2	NA
	Low	18.2	20.7	21.0	
MRT_last_ (h)	High	17.3	18.6	21.6	NA
	Low	17.8	19.3	20.3	
T_max_ (h)	High	12	12	12	1.000
	Low	12	12	12	

*PK-parameters area under the curve from the time of dosing extrapolated to infinity (AUC_inf_), area under the curve from the time of dosing to the last sampling time point (AUC_0~48_), area under the curve from the time of dosing to the last measurable positive concentration (AUC_last_), area under the first moment curve from dosing to the last measurable concentration (AUMC_last_), maximum observed concentration (C_max_), mean residence time from the time of dosing to the last measurable concentration (MRT_last_), time to the maximum concentration (T_max_), terminal half-life (T_1/2_λz), and elimination rate (λz). *Groups* control—healthy control calves, and BRD—*M. haemolytica* infected calves. *NA* not applicable.

**Mann–Whitney test was used to compare the high and low dose groups.

**Table 6:** The fecal pharmacokinetics of ciprofloxacin in calves administered enrofloxacin subcutaneously (low dose 7.5 mg/kg, high dose 12.5 mg/kg body weight, 14 animals in each healthy and BRD combined group) computed by a non-compartmental model (i.e., statistical moments) analysis.

Parameters*	Group	Minimum	Median	Maximum	Adjusted P-value**
AUC_inf_ (h × μg/mL)	High	381	424	467	0.533
	Low	325	375	426	
AUC_inf_/Dose	High	31	34	37	0.267
	Low	43	50	57	
AUC_0~48_ (h × μg/mL)	High	371	398	425	0.050
	Low	316	355	395	
AUC_last_ (h × μg/mL)	High	371	384	398	0.035
	Low	316	355	395	
AUMC_last_ (h^2^ × μg/mL)	High	6846	7368	7889	0.094
	Low	6633	7483	8333	
C_max_ (μg/mL)	High	18.2	21.1	24.1	0.062
	Low	13.7	18.5	23.3	
T_1/2_ λz (h)	High	6.4	8.4	10.5	NA
	Low	6.3	8.2	10.1	
λz (1/h)	High	0.07	0.09	0.11	NA
	Low	0.07	0.09	0.11	
MRT_inf_ (h)	High	22.2	22.3	22.3	NA
	Low	22.0	23.1	24.1	
MRT_last_ (h)	High	17.2	19.2	21.3	NA
	Low	21.0	21.1	21.1	
T_max_ (h)	High	8.0	10.0	12.0	0.347
	Low	12.0	12.0	12.0	

*PK-parameters area under the curve from the time of dosing extrapolated to infinity (AUC_inf_), area under the curve from the time of dosing to the last sampling time point (AUC_0~48_), area under the curve from the time of dosing to the last measurable positive concentration (AUC_last_), area under the first moment curve from dosing to the last measurable concentration (AUMC_last_), maximum observed concentration (C_max_), mean residence time from the time of dosing to the last measurable concentration (MRT_last_), time to the maximum concentration (T_max_), terminal half-life (T_1/2_λz), and elimination rate (λz). *Groups* control—healthy control calves, and BRD—*M. haemolytica* infected calves. *NA* not applicable.

**Mann–Whitney test was used to compare the high and low dose groups.

Most PK parameters, such as AUC_las_, C_max_, T_max_, T_1/2_λz, and λz, were not significantly different between healthy and sick calves in the enrofloxacin study. The exposure of both enrofloxacin (AUC_inf_/Dose: high dose 92 ± 24, low dose 98 ± 14, adj. *P* = 0.211) and ciprofloxacin (AUC_inf_/Dose: high dose 34 ± 5, low dose 50 ± 10, adj. *P* = 0.267) were not significantly different between the high dose and the low dose groups. However, the C_max_/Dose was higher in the low dose than the high dose for both enrofloxacin (high dose 4.9 ± 1.1, low dose 5.0 ± 1.2, adj. *P* = 0.015) and ciprofloxacin (high dose 1.7 ± 0.3, low dose 2.5 ± 0.9, adj. *P* = 0.041). The time to the maximum concentrations was not significantly different between the low and high-dose cohorts for both enrofloxacin and ciprofloxacin.

Danofloxacin had significantly higher total plasma concentration, peak concentration, and elimination half-life (AUC_last_ 21 ± 4, C_max_ 4 ± 0.7, T_1/2_λz 8 ± 1) compared to enrofloxacin (AUC_last_ 8 ± 3, C_max_ 0.7 ± 0.2, T_1/2_λz 5 ± 1) and ciprofloxacin (AUC_last_ 7 ± 3, C_max_ 0.5 ± 0.2, T_1/2_λz 5 ± 1). However, the time to the maximum concentration (4 ± 0.7), mean residence time (5 ± 0.4), elimination rate (0.09 ± 0.01), apparent clearance (0.4 ± 0.1), and apparent distribution (4.4 ± 1) were lower than that of enrofloxacin (T_max_ 5 ± 1, MRT 9 ± 1, λz 0.16 ± 0.04, Cl/F 1.2 ± 0.4, Vz/F 8.0 ± 3.4) and ciprofloxacin (T_max_ 6 ± 1, MRT 11 ± 1, λz 0.10 ± 0.02, Cl/F 1.3 ± 0.2, Vz/F 11 ± 3).

Similarly, the fecal PK values show that danofloxacin had higher total fecal concentration, peak concentration, and elimination half-life (AUC_last_ 3040 ± 451, C_max_ 334 ± 121, T_1/2_λz 10 ± 1) compared to enrofloxacin (AUC_last_ 920 ± 301, C_max_ 49 ± 17, T_1/2_λz 7 ± 1) and ciprofloxacin (AUC_last_ 370 ± 38, C_max_ 20 ± 5, T_1/2_λz 8 ± 2). Danofloxacin had smaller time to the peak concentration (10 ± 2), and the mean residence time (15 ± 3), elimination rate (0.07 ± 0.02) than that of enrofloxacin (T_max_ 12 ± 0, MRT 19 ± 2, λz 0.11 ± 0.02) and ciprofloxacin (T_max_ 11 ± 2, MRT 20 ± 2, λz 0.09 ± 0.02). Comparison of the plasma and fecal PK parameters show that danofloxacin had 145-fold higher concentration in feces than in plasma. Similarly, enrofloxacin and ciprofloxacin had 115- and 53-times higher concentrations in feces than in plasma, respectively.

## Discussion and conclusions

Enrofloxacin and danofloxacin are FQ drugs characterized by low toxicity and high efficacy against susceptible respiratory infections with excellent tissue penetration capability^19,26^. However, the increasing concerns about the effects of parenterally administered drugs in general and FQ drugs in particular on gastrointestinal microbes and their resistome as well as on the development of AMR in opportunistic and foodborne pathogens necessitate assessing pharmacokinetics of antibiotics used in disease prevention and control in livestock. With their excellent intestinal tissue penetration efficacy, FQs and their metabolites can be deposited in the intestinal lumen and induce undesired effects on animal and human health^13,14^. This situation warrants evaluating the dosing regimens of FQs that are used in animals through studying their pharmacokinetics. This study assessed and compared the plasma and fecal pharmacokinetics of danofloxacin, enrofloxacin, and its active metabolite ciprofloxacin in two to four months old calves. The main findings include significant PK differences between danofloxacin vs enrofloxacin and ciprofloxacin in both plasma and feces, between plasma and feces for both danofloxacin and enrofloxacin, as well as between low and high doses of enrofloxacin in both plasma and feces.

The plasma PK parameters of danofloxacin, enrofloxacin, and ciprofloxacin were not significantly different between healthy and *M. haemolytica* infected calves. A significant difference was reported previously in ducks^18^, concurrently infected pigs^27^, and calves^19^. It is known that infections alter the physiological status of animals; in particular, pathological changes in the liver and kidneys affect drug metabolism and clearance significantly^28^. For instance, in *Pasteurella multocida* infected ducks, the elimination period was prolonged, and the plasma concentration of danofloxacin increased compared to healthy ducks^18^. In the present study, the *M. haemolytica* infection caused only mild to moderate signs, and a week later, when the FQ drugs were administered, the calves had already recovered from the illness despite being more frequent and severe lung lesions were observed in the BRD groups up on necropsy one week later. BRD is a multifactorial disease, and the interactions among host, environment, and pathogen determine the severity of the disease that develops in an infected animal. Shipping of young animals and unfavorable weather conditions are often associated with BRD outbreaks on farms^29^. Future studies should aim at inducing relatively more severe disease as well as shortening the time between the challenge and the antibiotic administration. Nevertheless, it should be noted that FQs are labelled for administration to at-risk youngstock during outbreaks, meaning the infection/disease status of the study calves in the present study was similar to the disease status of those animals.

The dose normalized plasma concentration of danofloxacin was higher than that of enrofloxacin and ciprofloxacin, while its T_max_ and MRT were shorter. The total plasma concentration of danofloxacin (AUC) and the half-life time in this study are consistent with a study conducted in steers, while the time to the maximum drug concentration (T_max_) was extended in our study^14^. The differences that we observed between FQ drugs in the present study are consistent with a previous study, where the PK of danofloxacin and enrofloxacin were compared in 72 calves. Two single doses of danofloxacin (6 and 8 mg/kg) and a single dose of enrofloxacin (8 mg/kg) were administered to the calves subcutaneously. The concentration and time to the maximum concentration in plasma and various respiratory tissues were measured. The C_max_ and AUC_0~12_ were significantly higher for danofloxacin than enrofloxacin and ciprofloxacin; T_max_ and the half-life time were shorter in the danofloxacin group^5^. Direct comparisons of the values of the PK parameters between our findings and the study by TerHune and colleagues^5^ are difficult since they completed their study 12 h after drug administration, while our sampling extended up to 48 h; however, the values of the PK parameters are consistent between studies. Furthermore, the PK parameters of danofloxacin were comparable with a recent study conducted in calves, except for the significantly lower half-life in our study^19^. It is worthwhile to mention that danofloxacin mainly undergoes hepatic elimination while enrofloxacin undergoes renal elimination^2^, and that danofloxacin has a relatively high bioavailability^30^. In addition, the difference in the disposition of these antibiotics could be attributed to their difference in octanyl:water and tissues:plasma partition coefficients, describing the lipophilicity of drug that affect absorption and distribution^31–33^.

In contrast to our findings, a study conducted in premature calves did not report a higher plasma concentration of danofloxacin compared to enrofloxacin and its metabolite, which might be attributed to the difference in the ages of the calves and route (subcutaneous vs. intravenous and intramuscular) of drug administration^30^. Some PK parameters were shown to be significantly different between one-day and one-week-old calves^34^ as well as between three-weeks and six-month old calves^19^. The pharmacokinetics of drugs appear to be affected by the developmental stage of organs involved in the metabolisms and clearance of drugs, body compositions, amount of drug-binding proteins, etc., all of which are a function of age. For instance, metabolic enzymes such as cytochrome P-450 are not mature during early life, and thus the metabolism rate is slower in young animals compared to adults^35,36^.

Enrofloxacin undergoes de-ethylation and is metabolized to ciprofloxacin in the body. In the present study, the ciprofloxacin concentrations accounted for 44% and 51% of the total FQ concentrations in the low dose (7.5 mg/kg) and the high dose (12.5 mg/kg) groups, respectively. In contrast, the proportions of the ciprofloxacin concentrations in steers were 29% and 27% of the total FQ concentrations in single (12.5 mg/kg) and multiple (5.0 mg/kg for three injections) enrofloxacin regimens, respectively^37^. Furthermore, the enrofloxacin PK parameters were similar between the present study and the single dose enrofloxacin administered steers except for the C_max_ (1.21 ± 0.62 in steers, and 0.8 ± 0.23 in calves). In contrast, we observed higher values for most ciprofloxacin PK parameters, such as AUC_last_, AUC_inf_, C_max_, and elimination rate compared to the study in the steers. At the same time, the half-life and MRT were lower, and T_max_ was similar to that of Ferguson et al.^37^. The discrepancies in the ciprofloxacin PKs might be attributed to the difference in the age of the study animals^38^. Consistent with the present study, it was previously reported that the T_max_ and MRT were longer in one-day-old calves than one-week-old calves; however, unlike our observation, C_max_ was higher in older than in younger calves^34^. Similarly, a significantly higher AUC and lower clearance rate were observed in three-week-old calves than six-month-old calves^19^.

In this study, FQ and metabolite concentrations are several folds higher in the feces than in the plasma. Consistent with these observations, high concentrations of FQs in intestinal tissues were reported previously^3,14,37^. For instance, in the study conducted in dairy cattle, the concentration of danofloxacin in colonic tissues was 19 times higher than the concentration in the plasma; furthermore, the volume of distribution and the elimination rates were significantly higher in the tissues^3^. PK parameters such as AUC, C_max_, and MRT increased in intestinal tissues compared to plasma in steers, but the elimination rates and half-life were indifferent^37^. Multiple factors contribute to the higher concentrations of FQs in the intestinal tissues and the lumen (feces). FQ drugs have a high intestinal penetration rate from plasma^14,39^. In calves, 54% of enrofloxacin and 81% of ciprofloxacin concentration in the plasma were free, implying they could be secreted into the intestinal tissues and lumen without much hindrances^39^. Furthermore, active secretion and enterohepatic circulation have been shown to increase the concentrations of FQs, such as enrofloxacin and ciprofloxacin, in the intestinal lumen^13,40^. Danofloxacin has a higher concentration in the feces than enrofloxacin and ciprofloxacin with a shorter MRT, which might be attributed to differences in their disposition, including mechanisms of elimination and protein binding^31–33^.

The high concentrations of FQs in the intestinal lumen have practical implications; they affect opportunistic and foodborne pathogens residing in the gastrointestinal tract, disrupt microbial integrity, and induce selective pressure. For instance, the dosing regimens recommended for respiratory infections have been found to affect intestinal microorganisms in pigs^13,41^ and cattle^14,37^. Subcutaneously administered enrofloxacin to calves achieved the intestinal concentration that could decrease intestinal bacteria^42^. Further, our metagenomic studies^21,22^ have revealed that the fecal microbial diversity has been significantly changed in calves; some bacterial taxa responded differently to the low and high enrofloxacin doses. The fecal resistome profiles have been affected remarkably in FQs administered groups compared to the control groups^21,22^.

In closing, the plasma and fecal PKs of danofloxacin and enrofloxacin, along with its active metabolite ciprofloxacin, were analyzed for healthy and post-BRD challenge young calves. In summary, (1) BRD was induced in most of the calves infected with *M. haemolytica*; however, it was not severe enough to affect the plasma PKs of the FQs under investigation, while fecal concentrations of danofloxacin were significantly affected. Failure to induce severe disease and delaying of FQ administration following the BRD induction are the limitations of this study. However, future study should target administering FQs during the peak of the disease, which is two to four days following *M. haemolytica* inoculation*.* (2) The PK parameters of danofloxacin were significantly different from that of enrofloxacin and ciprofloxacin. The dose normalized plasma concentration of danofloxacin was two and three-fold higher than that of enrofloxacin and ciprofloxacin, respectively. Danofloxacin reached the peak concentration fast; it had a significantly shorter mean residence time, longer elimination half-life, slower elimination rate, smaller apparent clearance and distribution than enrofloxacin and ciprofloxacin. (3) The fecal concentrations of danofloxacin, enrofloxacin, and ciprofloxacin were several folds higher than the corresponding plasma concentrations. Furthermore, danofloxacin had three- and eight-fold higher concentrations than enrofloxacin and ciprofloxacin in the feces, respectively. However, since the fecal volume was not measured during sample collection, the cumulative amount of FQs excreted in the feces was not calculated. In conclusion, our findings show that subcutaneously administered danofloxacin and enrofloxacin expose fecal microbiota to high concentrations of FQs, which might cause selective pressure and change the microbial diversity and resistome. Furthermore, the higher fecal concentration of danofloxacin coupled with its slower elimination rate and longer half-life call for a further investigation if this antibiotic plays a more significant role than enrofloxacin in causing alteration of gut microbiota and its resistome. In general, this study provides comprehensive information on the plasma and fecal pharmacokinetics of FQs used in food animals.
